# A Preliminary Study of Swell-Drying as an Innovative Process for Improving the Nutritional Quality of Dried Lucuma (*Pouteria lucuma*) and Dried Goldenberry (*Physalis peruviana* L.)

**DOI:** 10.3390/foods14203477

**Published:** 2025-10-12

**Authors:** Carmen Téllez-Pérez, Maritza Alonzo-Macías, Colette Besombes, Gastón Cruz, Daniel Marcelo-Aldana, Antonio Rodriguez-Zevallos, Karim Allaf, Anaberta Cardador-Martínez

**Affiliations:** 1 Laboratory of Engineering Science for Environment LaSIE-UMR-CNRS 7356, Eco-Intensification of Agro-Industrial Eco-Processes, La Rochelle University, 17042 La Rochelle, France; ctellezperez@gmail.com (C.T.-P.); colette.besombes@univ-lr.fr (C.B.); karim.allaf@univ-lr.fr (K.A.); 2 Tecnologico de Monterrey, Escuela de Ingeniería y Ciencias, Epigmenio González 500, San Pablo 76130, Querétaro, Mexico; malonzoma@tec.mx; 3 Facultad de Ingeniería, Universidad de Piura, Av. Ramon Mugica 131, Piura 20009, Peru; gaston.cruz@udep.edu.pe (G.C.); daniel.marcelo@udep.edu.pe (D.M.-A.); 4 Escuela de Ingeniería en Industrias Alimentarias, Facultad de Ciencias Agrarias, Universidad Privada Antenor Orrego, Av. América Sur 3145, Urb. Monserrate, Trujillo 13008, Peru; arodriguezz@upao.edu.pe

**Keywords:** interval drying, instant controlled pressure drop, *Pouteria lucuma*, *Physalis peruviana* L., antioxidants and nutritional components

## Abstract

Lucuma and goldenberry are rich in bioactive compounds, and swell-drying (SD) can help preserve these properties. This study examined how SD impacts the nutritional quality of lucuma and goldenberry. The SD process involved the following: (1) initial pre-drying, with Interval Highly Active Drying (IHAD) for lucuma and Continuous Convective Airflow Drying (CCAD) for goldenberry, (2) a DIC treatment under an experimental design with 13 treatments, and (3) a final CCAD step. The parameters studied for DIC were steam pressure (0.1 to 0.5 MPa) and treatment time (5 to 55 s). Bromatological analysis and antioxidant activity were the response variables. Under accurate SD conditions, both fruits maintained their nutritional quality and increased their antioxidant activity compared to controls. Carbohydrates, proteins, lipids, fiber, and ash average contents of lucuma were 88.73%, 7.28%, 1.18%, 1.88%, and 0.92%, respectively. The DIC treatment of 0.27 MPa for 22 s increased the percentage of ABTS and the DPPH inhibition of lucuma by 1.2 and 1.5 times, respectively. For goldenberry, carbohydrates, proteins, lipids, fiber, and ash average contents were 71.87%, 7.18% 7.01%, 6.60%, and 6.77%, respectively. DIC treatment of goldenberry at 0.5 MPa for 30 s increased ABTS % inhibition by 1.5 times, and DIC at 0.10 MPa for 30 s increased DPPH inhibition by 4.9%.

## 1. Introduction

The Andean region has a wide variety of native species such as lucuma (*Pouteria lucuma*) and goldenberry (*Physalis peruviana* L.) [1,2]. Lucuma is a perennial fruit native to Peru, Chile, and Ecuador. It is widely used in the food industry in both fresh and dried forms to make confections, yogurt, ice cream, baby food, pastries, and baked goods [1,3]. This fruit is characterized by its antioxidant compounds, such as carotenoids and polyphenols [4,5,6]. When ripe, the pulp accounts for 69–82% of the fruit, while the peel and seeds make up 18–31%. Fresh lucuma pulp is bright yellow, dry, powdery, and sweet, with a moisture content ranging from 58 to 72% on a wet basis.

On the other hand, the goldenberry, better known in Peru as aguaymanto, is a tropical fruit characterized by a sweet and sour flavor and a rich content of bioactive compounds (i.e., carotenoids, phytosterols, and polyphenols) [2]. In fact, thanks to its nutraceutical and pharmaceutical properties, it has been considered a “superfood” and is widely used in both fresh and dried states as a medicinal fruit [7,8]. At ripeness, the moisture content of fresh fruit ranges from 76 to 85% w.b. [9].

Since maintaining fruit quality is a major challenge in the fresh supply chains of lucuma and goldenberry, the dried form is the most common presentation of these fruits on the global market [5,10,11]. In fact, to ensure optimal product quality and shelf life, the final moisture content should be less than 10% (w.b.), and the optimal storage water activity should be between 0.2 and 0.4 [1,3,5,9,12]. Then, to reduce moisture content to safe levels for storage, various drying technologies such as solar drying, airflow drying, vacuum drying, and freeze drying have been used [13,14]. However, none of these drying techniques guarantees the elimination of vegetative bacteria, spore-forming bacteria, and insects (which sometimes are present in solar-dried products). In this regard, the instant controlled pressure drop technology (DIC) becomes a vital tool for improving the overall quality of dried food.

DIC is a thermo-mechanical process in which food matrices are exposed to saturated steam pressure treatments (100 to 900 kPa) for a short period (a few seconds), followed by an instant controlled pressure drop to a final vacuum of 10 to 5 kPa. This abrupt change in pressure and temperature causes immediate autovaporization of water and quick cooling of the food. It is influenced by the rheological properties of the food matrix and cell expansion [15]. Several studies have demonstrated that thermal and mechanical mechanisms can effectively inactivate bacteria in both vegetative and spore forms [16,17,18]. Additionally, this technology has increased the availability of bioactive compounds in various dried vegetables and fruits such as apples [19], strawberries [20], beetroots [21], and peppers [22], among others. Consequently, combining airflow drying with DIC technology has been called “swell-drying,” which enhances the overall quality of dried food products.

Swell-drying is a hybrid dehydration process that combines three stages: (1) an initial partial drying step to achieve a moisture content between 15 and 30% w.b., with this initial moisture level varying depending on the rheological properties of the food product (food matrix composition, glass transition temperature, and viscoelasticity of the cell wall); (2) one DIC treatment step; and (3) a second drying step to reach a final moisture content below 10% w.b. Various studies have shown that when the glass transition temperature is crossed, swell-drying improves the drying rate, enhances porosity and texture, and improves the organoleptic and nutritional characteristics of dried products, such as color, nutrient retention, and antioxidant activity [23,24,25]. In fact, the expanded microstructure increases effective moisture diffusivity during subsequent drying, reduces shrinkage and collapse, and improves rehydration and textural attributes (e.g., crispness). Furthermore, the enhanced porosity also facilitates solvent access and often increases the extractability of bioactive compounds such as antioxidants [15,26,27,28].

On the other hand, although continuous convective airflow drying (CCAD) is the most common drying technique in food industries, conventional drying systems are often characterized by low energy efficiency and may induce thermal degradation of sensitive nutrients [29]. In fact, during CCAD, only a small fraction of the supplied heat is actually used to evaporate the water inside the food, and most of the thermal energy is lost in heating up the airflow and the dryer. Allaf et al. [30] defined three main stages during convective air drying: (1) starting accessibility stage, (2) diffusion stage, and (3) paradoxical stage. During the starting accessibility stage, water evaporates from the product surface. Then, under adequate airflow, temperature, and relative humidity, the external resistance becomes negligible. At the diffusion stage, heat and mass transfer are slow because moisture must migrate from the food core to the surface by diffusion. Thus, when the external resistance becomes negligible, internal diffusivity becomes the limiting phenomenon. The paradoxical stage is characterized by a drying through “front progression” kinetics, driven by a higher vapor pressure at the surface compared to the core.

Therefore, when seeking new alternatives that use minimal energy while preserving food bioactive components, Interval Highly Active Drying (IHAD) emerges as an interesting drying process to be combined with the instant controlled pressure drop technology (DIC). Interval Highly Active Drying (IHAD) involves separating the heat and mass transfer stages at the surface of the food product (active period) from the mass transfer stage inside the product (tempering time) [30]. In the case of interval highly active airflow drying, the process alternates short active drying periods with hot airflow to facilitate the evaporation of surface water (t_ON_ varies from less than one second to a few seconds) and tempering intervals with no airflow to allow internal water to diffuse and reach the exchange surface (t_OFF_ ranges from a few seconds to several minutes) [31,32]. This method improves drying efficiency and preserves product quality in fruits and heat-sensitive items. Thus, by coupling IHAD and DIC treatment, innovative SD processing could be defined.

In this study, the effects of swell-drying on the nutritional quality of lucuma and goldenberry were investigated. The swell-drying of fresh lucuma was performed by initially applying a pre-drying step using Interval Highly Active Drying, followed by an experimental design with 13 DIC treatments, and concluding with a final drying step using continuous airflow drying. Likewise, the swell-drying of goldenberry involved the use of a previously pre-dried commercial product, followed by an experimental setup with 13 DIC treatments, and a final phase of continuous airflow drying. Goldenberry and lucuma samples exclusively dried under continuous convective air drying (CCAD) were used as controls. To assess the impact of DIC treatment, saturated steam pressure and thermal treatment time were used as independent variables, while bromatological analysis and antioxidant activity served as response variables.

## 2. Materials and Methods

### 2.1. Materials

#### 2.1.1. Biological Materials

A total of 3 kg of fresh and fully ripe *Pouteria lucuma* fruits was purchased at a supermarket in Piura, Peru, and 1 kg of pre-dried *Physalis peruviana* L. fruits was bought at the “La Viña Foods” company in Piura, Peru.

#### 2.1.2. Reagents and Solvents

Potassium persulfate, 2,2-diphenyl-1-picrylhydrazyl (DPPH), and 2,2′-azino-bis(3-ethylbenzothiazoline)-6-sulfonic acid (ABTS) were purchased from Sigma-Aldrich Canada Ltd. (Oakville, ON, Canada). The water used was obtained from a Millipore Milli-Q water system with a resistivity of 18.2 MΩ·cm (25 °C). All solvents used were HPLC-grade (Sigma-Aldrich, St. Louis, MO, USA).

### 2.2. Methods

**Figure 1:**
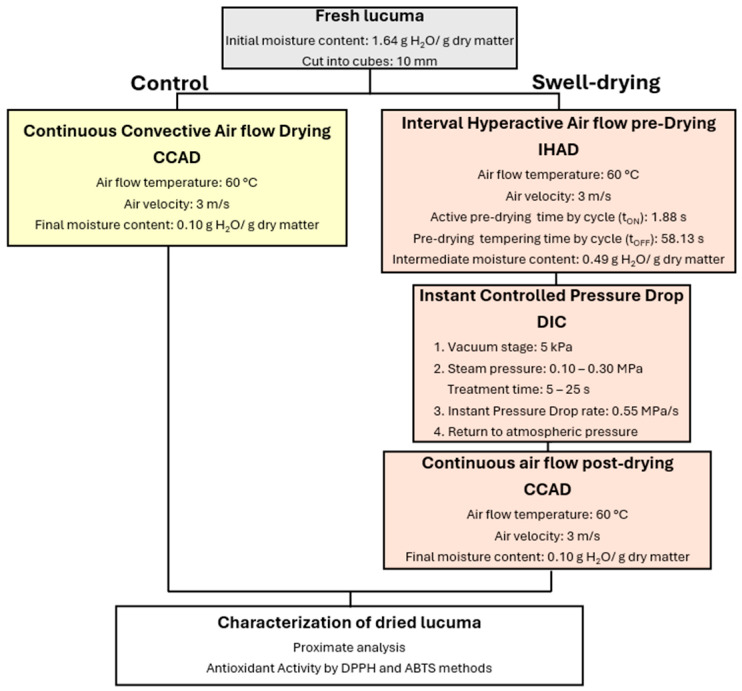
Drying protocol of fresh lucuma. Lucuma cubes exclusively dried under continuous convective air drying (CCAD) were used as a control. SD lucuma consisted of an initial pre-drying by IHAD, a DIC treatment, and a final CCAD post-drying.

**Figure 2:**
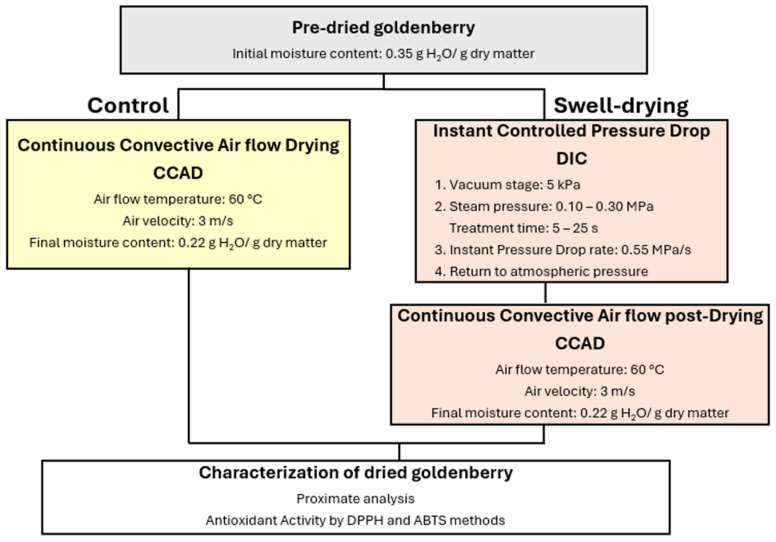
Drying protocol of goldenberry. Pre-dried goldenberries, exclusively dried under continuous convective air drying (CCAD), were used as a control. SD goldenberry consisted of applying DIC treatment to previously dried goldenberry, followed by a final CCAD post-drying.

The drying protocol of lucuma and goldenberry is shown in Figure 1 and Figure 2, respectively.

#### 2.2.1. Sample Preparation

**Figure 3:**
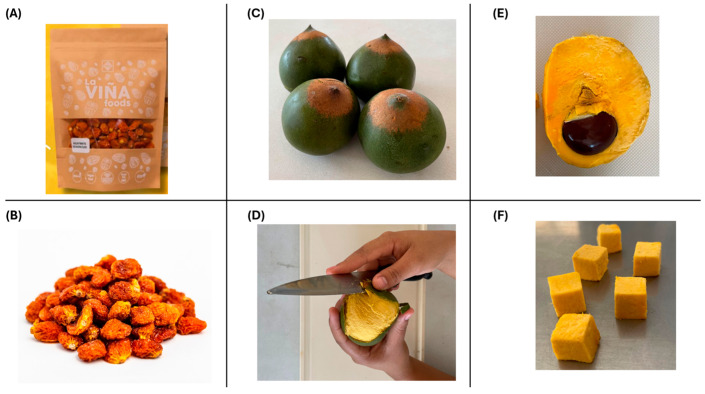
Goldenberry and lucuma samples. (**A**) Commercial presentation of pre-dried goldenberry; (**B**) pre-dried goldenberry; (**C**) fresh lucuma; (**D**) peeling lucuma; (**E**) pitting lucuma; and (**F**) cutting lucuma cubes.

Before DIC treatment, only the initial moisture content was determined for pre-dried golden berries. Figure 3A,B show the goldenberry samples in their commercial presentation. Conversely, for fresh lucuma (Figure 3C), the fruits were washed, manually peeled, and pitted (Figure 3D,E). Then, the pulp was cut into 10 mm cubes (Figure 3F), and the initial moisture content was measured.

#### 2.2.2. Continuous Convective Airflow Drying (CCAD) of Lucuma and Goldenberry

The goldenberry and lucuma samples exclusively dried under continuous convective air drying (CCAD) were used as controls. In total, 200 g of control samples was dried via continuous convective air drying at 60 °C with an airflow of 3 m/s. The total drying times of lucuma and goldenberry were 26 h and 44 h, respectively. The final moisture contents of control lucuma and goldenberry were 0.10 and 0.22 g H_2_O/g dry matter, respectively.

#### 2.2.3. Pre-Drying of Lucuma Using Interval Highly Active Drying (IHAD)

Fresh lucuma cubes were pre-dried using Interval Highly Active Drying at 60 °C, with an airflow of 3 m/s, a constant active time t_ON_ of 1.88 s, and a constant tempering time t_OFF_ of 58.13 s. The lucuma was dried by passing compressed hot air through two pneumatic valves: one distributing hot airflow perpendicular to the samples during t_ON_ and the other distributing hot airflow in open air during t_OFF_. Total active pre-drying time (t_ON_) was 4.38 min. Water evaporation was manually measured during the process. The average moisture content of the pre-dried lucuma was 0.49 g H_2_O per g of dry matter. After pre-drying, the samples were stored for 2 days at 4 °C.

#### 2.2.4. Determination of Moisture Content and Water Activity

Moisture content was determined by placing 1 g of fresh, pre-dried, or dried sample in an oven at 105 °C for 24 h (Memmert UF30, Schwabach, Germany) (AOAC 23.003:2003) [33]. To measure water activity (a_w_), the protocol of Yu and Schmidt [34] was adopted, and the AquaLab 4TE equipment (Decagon Device Inc., Pullman, WA, USA) was used. Water activity was measured only in controls and SD samples of lucuma and goldenberry corresponding to the central points. Measurements were performed in triplicate.

#### 2.2.5. DIC Treatment, Experimental Design, and Post-Drying

**Figure 4:**
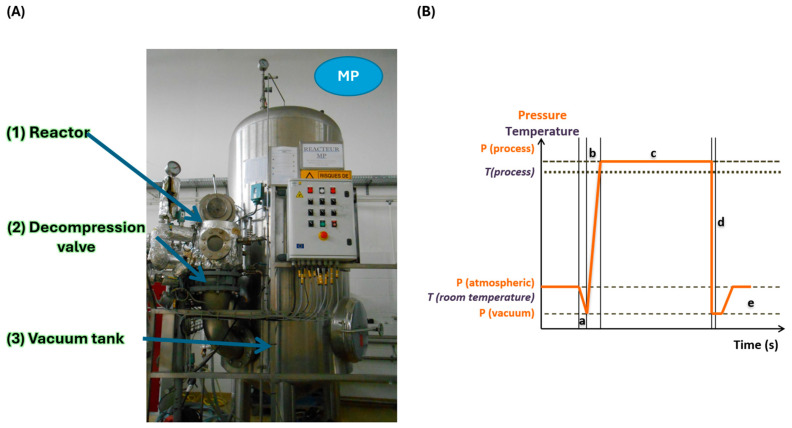
(**A**) Representation of DIC MP laboratory equipment: (1) reactor, (2) decompression valve, and (3) vacuum tank. (**B**) Schematic representation of a DIC processing cycle: (a) establishing the vacuum; (b) injecting saturated steam pressure; (c) maintaining the selected steam pressure during the specified thermal processing time; (d) instant controlled pressure drop towards the vacuum; and (e) stabilization at atmospheric pressure.

The DIC treatment of pre-dried goldenberry and lucuma was performed using the MP lab-scale DIC reactor (ABCAR-DIC Process, La Rochelle, France) (Figure 4A). This reactor has been described in previous studies [21,35,36], and its main components include a processing vessel (reactor) (1), an instantaneous decompression valve (2), and a vacuum tank (3).

The effects of DIC treatment on nutritional composition and antioxidant activity, evaluated by DPPH and ABTS assays, were studied using a central composite rotatable design. For both pre-dried fruits, saturated steam pressure (P) and thermal treatment time (t) were selected as independent variables. In addition, before defining the experimental design for lucuma and goldenberry, preliminary assays were carried out. In the case of lucuma, saturated steam pressure between 0.1 MPa and 0.4 MPa and a thermal treatment time between 10 and 40 s were evaluated. This first approach showed that under saturated *p* ≥ 0.4 MPa, samples changed their color from yellow to brown. On the other hand, in the case of goldenberry, preliminary assays were carried out between 0.1 and 0.6 MPa and for 10 to 55 s. Under *p* ≥ 0.6 MP, goldenberry changed its color from light brown to dark brown. For these reasons, by looking to preserve the most physico-chemical properties and to avoid severe thermal damage, the lucuma DIC experimental design was defined between 0.1 and 0.3 MPa and for 5–25 s, and the goldenberry experimental design was defined between 0.1 and 0.5 MPa and for 5–55 s. Table 1 details the experimental design for pre-dried lucuma, while Table 2 shows the design for pre-dried goldenberry.

The DIC treatment involved placing 60 g of pre-dried samples inside the reactor and establishing a vacuum of 30 mbar (Figure 4B-a). Next, saturated steam was injected into the reactor until the selected pressure (ranging from 0.1 to 0.5 MPa) (Figure 4B-b), which was maintained for a short period (from 5 to 55 s) (Figure 4B-c). Then, the samples were subjected to an instant controlled pressure drop (ΔP/Δt > 0.5 MPa·s^−1^) to vacuum (30 mbar) (Figure 4B-d). Finally, the pressure was released to atmospheric pressure (Figure 4B-e), and dried goldenberry and lucuma were recovered.

The design included 13 experiments with four factorial points (2^2^) (+1, +1; +1, −1; −1, −1; and −1, +1), four star points (−α, 0; +α, 0; 0, +α; and 0, −α), and five central points (0, 0). Experiments were conducted randomly. After DIC treatment, samples were post-dried via continuous convective air drying at 60 °C with an airflow of 3 m/s. The total post-drying times of lucuma and goldenberry samples were 17 h and 14 h, respectively. Finally, all treated and untreated samples were stored at room temperature (20–25 °C) until further analysis.

#### 2.2.6. Bromatological Analysis

Proximate analysis was performed on lucuma (*Pouteria lucuma*) and goldenberry (*Physalis peruviana* L.) using official AOAC methods [37,38]. The parameters measured included moisture content (method 930.15), protein (method 981.10), fat (method 920.39), and ash (method 942.05). Carbohydrates were calculated by subtracting lipids, proteins, dietary fiber, and ash from the total. Results were expressed on a dry matter basis.

#### 2.2.7. Antioxidant Analysis

##### Extract Preparation

DIC-treated lucuma and goldenberry fruit samples, along with control samples (raw material), were ground using a coffee grinder (Krups GX4100, Solingen, Germany) until a fine powder was achieved. One gram of each sample was weighed into a 15 mL conical tube and mixed with 10 mL of methanol. The samples were then macerated in the dark at room temperature (25 °C) for 24 h under orbital agitation at 150 rpm (Junior Orbit Shaker 3520, Chennai, India). After maceration, the samples were centrifuged at 4000 rpm for 10 min at 25 °C using a Heraeus Multifuge X1R centrifuge (Thermo Scientific, MA, USA). The supernatant was collected and stored at −20 °C for further analysis. All samples were prepared in duplicate.

##### ABTS Assay

Antioxidant activity was assessed by measuring the extracts’ ability to scavenge the ABTS^+^ radical cation (2,2′-azino-bis(3-ethylbenzothiazoline-6-sulfonic acid)), based on the method outlined by Nenadis and Wang [39], with minor modifications. A 2.45 mM potassium persulfate solution was prepared and kept in the dark at room temperature for 24 h. Then, a 7 mM ABTS solution was mixed with the pre-activated persulfate solution and allowed to react in the dark for 30 min to generate the ABTS^+^ radical.

The resulting ABTS^+^ solution was diluted with ethanol until an absorbance of 0.8 ± 0.1 was achieved at 734 nm. For the assay, 40 µL of the sample was added to a 96-well microplate, followed by 200 µL of the ABTS^+^ solution. The mixture was incubated for 6 min with gentle shaking, and absorbance was measured using a UV-Vis microplate reader (xMark™, BioRad, Hercules, CA, USA). All measurements were conducted twice.

The free radical scavenging activity was determined by calculating the percentage inhibition using Equation (1):

(1) $$I\%\;by\;ABTS=\frac{A_{c}-A_{s}}{A_{c}}\%$$

where

*A_c_ =* absorbance of the control;
*A_s_ =* absorbance of the sample.

##### DPPH Assays

The free radical scavenging activity was also assessed using the DPPH method, following the protocols by Fukumoto and Mazza [40] and Sarker and Oba [41] with minor modifications. In brief, 40 µL of each extract was combined with 200 µL of a 250 µM DPPH solution (in methanol) in a 96-well flat-bottom microplate. After 30 min of incubation at room temperature in the dark, absorbance was measured at 517 nm with a spectrophotometer (xMark™, BioRad, CA, USA). Each test was conducted in triplicate.

The inhibition percentage was determined with Equation (2):

(2) $$I\%\;by\;DPPH=\frac{A_{c}-A_{s}}{A_{c}}\%$$

where

*A_c_ =* absorbance of the control;
*A_s_ =* absorbance of the sample.

#### 2.2.8. Statistical Analysis

Statgraphics Plus software version XVI (Statgraphics Technologies Inc., The Plains, VA, USA) was used for statistical analysis. For the experimental design of DIC treatment, statistical analysis was performed using analysis of variance, Pareto charts, and response surface methodology. Analysis of variance (ANOVA) was used to test the statistical significance of steam pressure (MPa) and thermal treatment time (s) factors on the response variables: moisture (%), carbohydrates (%), lipids (%), proteins (%), fiber (%), ashes (%), and antioxidant activity measured by DPPH and ABTS methods (% inhibition). In this study, a significance level of 0.05 was used. The Pareto chart was used to visualize the magnitude and impact of different treatment effects. The vertical line in the Pareto chart indicates the effects that are statistically significant at the 95% confidence level. Response surface methodology was employed to fit the response to a second-order (quadratic) polynomial equation and to determine the predicted optimal input settings of *P* and *t* to maximize each response. In this preliminary research, the experimental validation of the predicted optimal operating conditions, the modeling validation, and the goodness of fit were not assessed.

## 3. Results and Discussions

### 3.1. Raw Material Characterization

**Figure 5:**
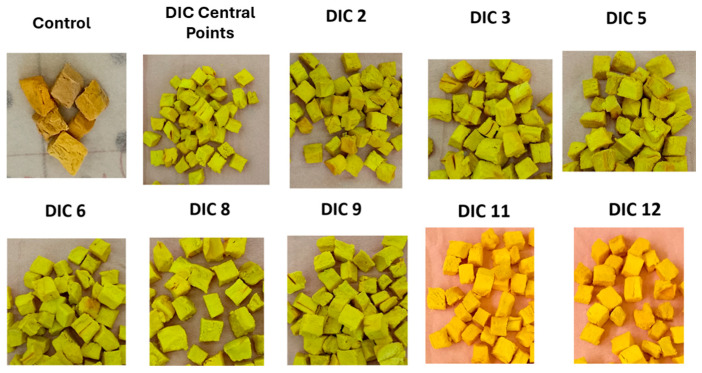
Appearance of dehydrated lucuma: control and swell-dried samples. Control (untreated sample), DIC central points (0.20 MPa, 15 s), DIC 2 (0.30 MPa, 15 s), DIC 3 (0.20 MPa, 25 s), DIC 5 (0.27 MPa, 22 s), DIC 6 (0.27 MPa, 8 s), DIC 8 (0.13 MPa, 8 s), DIC 9 (0.13 MPa, 22 s), DIC 11 (0.10 MPa, 15 s), and DIC 12 (0.20 MPa, 5 s).

After peeling and pitting, the percentage of high-quality lucuma pulp was 74%, while peel and stone waste accounted for 18% and 8%, respectively. Additionally, the initial moisture content of lucuma pulp was 62.12% on a wet basis or 1.64 g H_2_O/g solids on a dry basis. This result aligns with those of Aguilar-Galvez and García-Ríos [1], who indicated that pulp makes up 69–82% of the fruit, and its moisture content ranges from 64 to 72%. Figure 5 shows the appearance of the control and SD lucuma.

**Figure 6:**
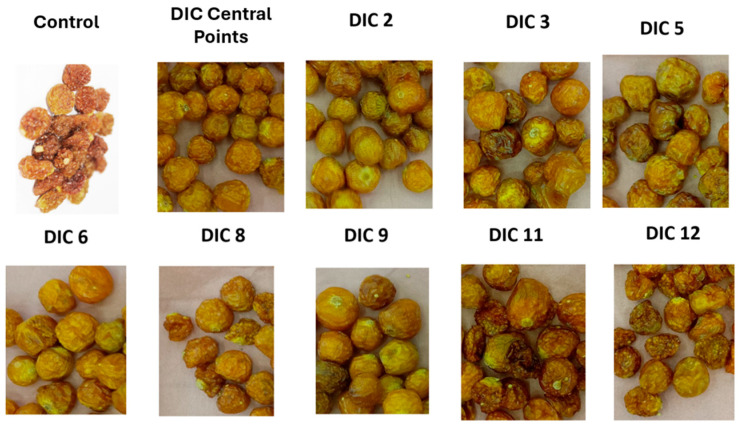
Appearance of dehydrated goldenberry: control and swell-dried samples. Control (untreated sample), DIC central points (0.30 MPa, 30 s), DIC 2 (0.50 MPa, 30 s), DIC 3 (0.30 MPa, 55 s), DIC 5 (0.44 MPa, 48 s), DIC 6 (0.44 MPa, 12 s), DIC 8 (0.16 MPa, 12 s), DIC 9 (0.16 MPa, 48 s), DIC 11 (0.10 MPa, 30 s), and DIC 12 (0.30 MPa, 5 s).

For goldenberry, the initial moisture content of commercial pre-dried fruit was 25.93% on a wet basis or 0.35 g H_2_O/g solids on a dry basis. According to Yıldız and İzli [42], the dry matter of fresh goldenberry fruit was 18.67%, which means that to produce 1 kg of pre-dried goldenberry, approximately 4 kg of fresh fruit needs to be dried. Figure 6 shows the appearance of the control and SD goldenberry.

### 3.2. Bromatological Analysis Results

Table 3 displays the bromatological analysis of dried lucuma. As previously noted, the initial moisture content of fresh lucuma pulp was 62.12% w.b., and after drying, the final moisture content of all dried lucuma samples was 9.7% w.b. In fact, the final moisture content ranged from 7.98% to 11.8%. According to the study by Erazo, Escobar [4], the moisture content of fresh lucuma (six varieties) ranged from 56.03% to 63.16% w.b. Similar results were reported by Maza-De la Quintana and Paucar-Menacho [10], who observed moisture content in fresh lucuma between 62% and 72.3%. Regarding the final moisture content of dried lucuma, the study by Barrena Gurbillón and Quintana [43] indicated that the equilibrium moisture content was 3.85% w.b. or 0.04 g H_2_O/g dry matter. In this study, the goal was not to reach equilibrium moisture content but to achieve a safe storage moisture level. Typically, moisture levels in commercial products like lucuma flour or powder are kept below 10% for stability [44]. Furthermore, the water activity of the control samples was 0.374 ± 0.011, while the a_w_ of SD samples treated under DIC central points was 0.294 ± 0.018. These results provide supplementary information on the stability of SD products. Previous studies have shown that the new expanded structure obtained by the DIC process increases the adsorption capacities of dried products, which directly impacts the water activity reduction [35,45,46].

Furthermore, Table 3 shows the bromatological analysis of dried lucuma. In fact, the average macronutrient contents for control and swell-dried samples for carbohydrates, proteins, lipids, fiber, and ash of lucuma were 88.73%, 7.28%, 1.18%, 1.88%, and 0.92%, respectively. The lipid content in dried lucuma ranged from 1.16 to 1.46% of dry matter. These results align with those from studies by Maza-De la Quintana and Paucar-Menacho [10], and Erazo and Escobar [4], which reported lipid contents between 0.52 and 2.17% of dry matter. Regarding the protein content in dried lucuma from this study, it ranged from 7.42 to 9.01%. In contrast, the studies by Maza-De la Quintana and Paucar-Menacho [10], as well as Erazo and Escobar [4], showed lower protein levels, between 4.81 and 6.05% of dry matter. Additionally, fiber content ranged from 0.11 to 1.85%. Erazo and Escobar [4] reported a fiber range between 1.97 and 2.80%, whereas Maza-De la Quintana and Paucar-Menacho [10] found values from 2.89 to 26.63% of dry matter.

Likewise, the ash content of dried lucuma samples ranged from 1.29% to 2.42% of dry matter; in this regard, Erazo and Escobar [4] reported an ash range between 1.6% and 2.79% of dry matter. Finally, examining the carbohydrate content of lucuma samples, they varied between 86.72% and 91%. Maza-De la Quintana and Paucar-Menacho [10], as well as Erazo and Escobar [4], indicated similar results with a carbohydrate content between 87.29% and 91.12%.

**Figure 7:**
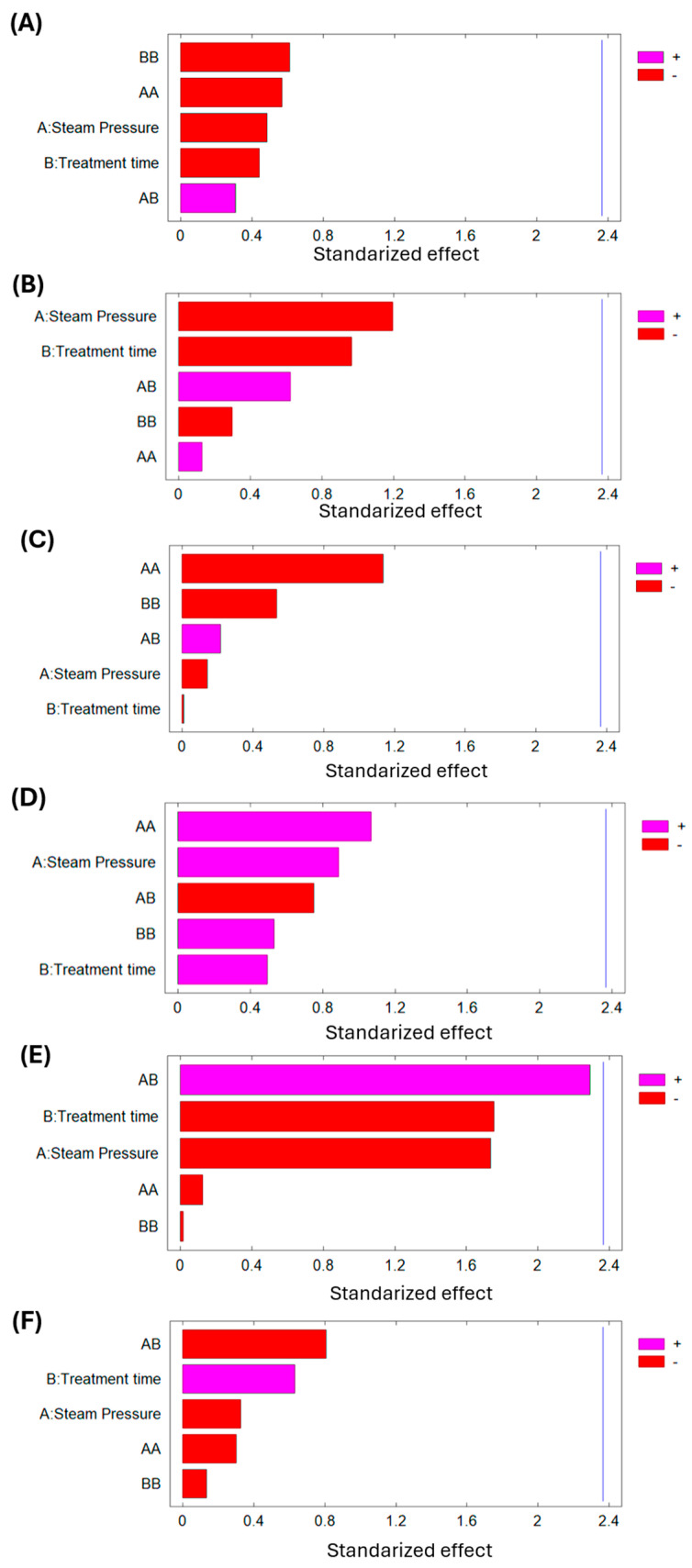
Effect of DIC treatment on (**A**) final moisture content; (**B**) lipid content; (**C**) protein content; (**D**) carbohydrate content; (**E**) fiber content; and (**F**) ash content of dried lucuma.

Additionally, Table 4 with the analysis of variance for bromatological analysis of SD lucuma and Figure 7 (Pareto chart) demonstrate that any of the studied DIC variables (P and t) significantly affect the final moisture content, lipid content, protein content, carbohydrate content, fiber content, and ash content of dried lucuma material.

The composition of lucuma varies according to genetic, environmental, and postharvest factors, which could explain the slight differences in lucuma’s bromatological composition between this study and the referenced literature. Lucuma varieties may differ in seed, skin, and pulp ratios. Furthermore, the ripening stage directly affects macronutrient levels, and postharvest handling can lead to enzymatic degradation of nutrients [1,47]. Therefore, selecting the optimal ripeness stage before drying is very important.

Table 5 presents the bromatological analysis of dried goldenberries and shows that, starting with an initial moisture content of 25.93% w.b. in pre-dried goldenberries, the DIC treatment and drying resulted in a final moisture content ranging from 9.61% to 22.95%. Nawirska-Olszańska and Stępień [7] noted that the thick, stiff, and waxy skin of goldenberry makes dehydration difficult because it acts as a barrier that limits water loss. To improve mass transfer through the skin, several methods have been studied, including physical pretreatments such as puncturing the peel with a penetrator [7], blanching at 96 °C for 25 min [48], and microwaving [7], as well as chemical pretreatments like applying 9.48% olive oil combined with 4.74% K_2_CO_3_ at 28 °C for 60 min [48]. In this study, the lowest moisture content (9.61%) of goldenberry was observed after a DIC treatment at 0.30 MPa for 30 s, suggesting that these conditions help promote moisture evaporation from inside the fruit. In addition, the water activity of control samples was 0.421 ± 0.013, while the a_w_ of SD samples treated under DIC central points was 0.373 ± 0.006. Dried fruits and powders often have final a_w_ values between 0.20 and 0.40, which ensures long shelf life if packaged to avoid moisture uptake and under temperatures around 20 °C [49,50].

On the other hand, Table 5 shows the bromatological analysis of dried goldenberry. The average carbohydrates, proteins, lipids, fiber, and ash of control and swell-dried goldenberry were 71.87%, 7.18% 7.01%, 6.60%, and 6.77%, respectively. The lipid content of dried goldenberry ranged from 5.25 to 8.84% of dry matter. These results are higher than those reported by Sierra and Escobar [51] and Campos and Chirinos [52], who noted a lipid content between 1 and 3% of dry matter. Regarding the protein content of dried goldenberry, it varied from 5.15 to 8.61% of dry matter. In this context, Sierra and Escobar [51] found a wide range of proteins, between 0.15 and 8% of dry matter. Additionally, for fiber, the values ranged from 2.48 to 8.25% of dry matter. Campos and Chirinos [52] reported a fiber content of 3.97% of dry matter. It should be noted that Siddiqui and Ucak [53] reported that higher drying temperatures can lead to nutritional losses of macronutrients. However, to the best of our knowledge, no studies have established how goldenberry fiber is affected when subjected to saturated steam pressure. Referring to Table 5, the fiber reduction observed in the DIC 3 goldenberry sample may be associated with the longest thermal treatment time applied (55 s), which could have triggered hydrolysis and structural fiber damage. Future studies should include additional trials to better clarify the impact of extended treatment times on goldenberry nutrients.

Furthermore, the ash content of goldenberry samples ranged from 5.97% to 7.34% of dry matter. In this regard, Campos and Chirinos [52] reported an ash content of 3.95% of dry matter. Lastly, concerning the carbohydrate content in goldenberry samples, it varied between 69.4% and 77.25% of dry matter. Sierra and Escobar [51] and Campos and Chirinos [52] indicated a carbohydrate content in goldenberry between 81% and 98% of dry matter.

**Figure 8:**
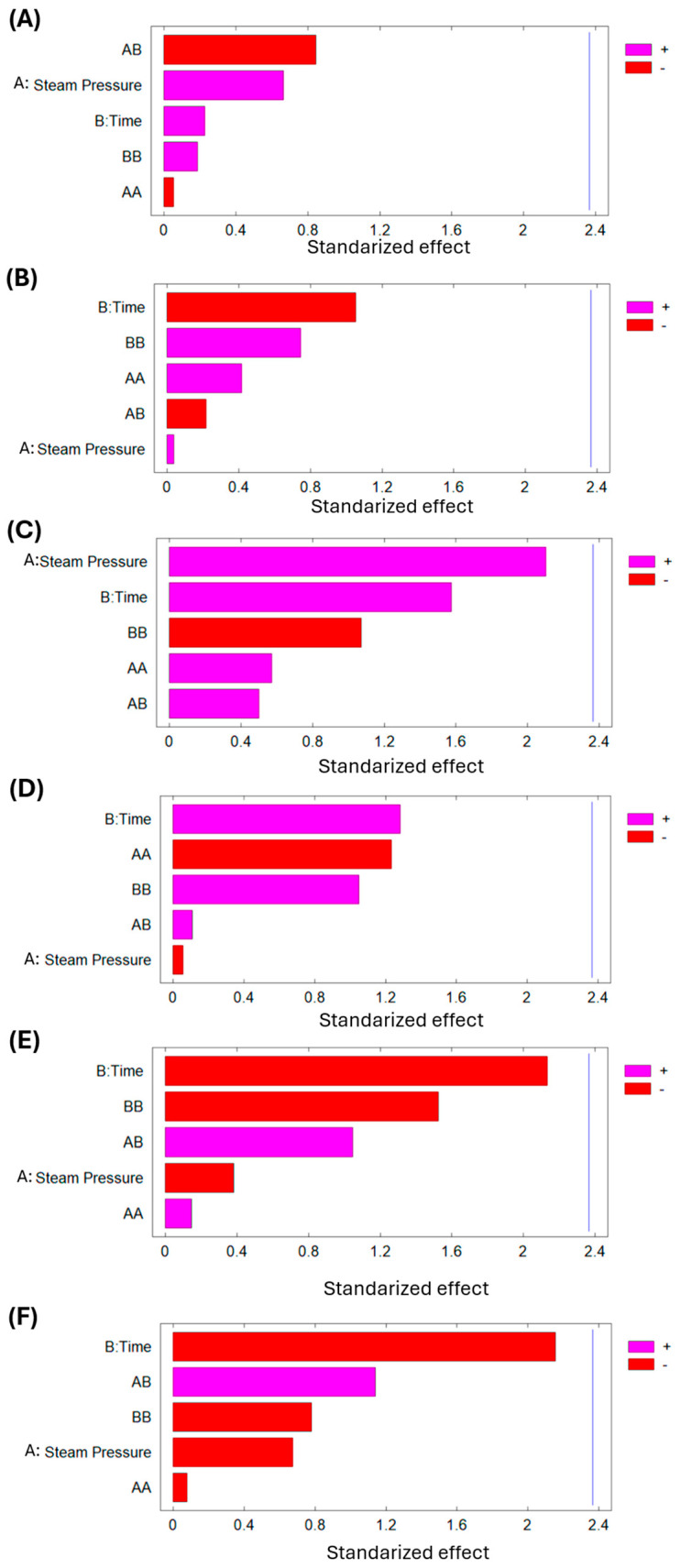
Effect of DIC treatment on (**A**) final moisture content; (**B**) lipid content; (**C**) protein content; (**D**) carbohydrate content; (**E**) fiber content; and (**F**) ash content of dried goldenberry.

In addition, Table 6 with the analysis of variance for bromatological analysis of SD goldenberry and the Pareto chart of Figure 8 demonstrate that any of the studied DIC variables (P and t) significantly affect the final moisture content, lipid content, protein content, carbohydrate content, fiber content, and ash content of dried goldenberry material. Numerical differences in macronutrient composition are negligible between controls and treated samples, which means that swell-drying does not affect the nutritional composition of goldenberry.

### 3.3. Antioxidant Activity

The antioxidant activity of dried lucuma, measured by the percentage of ABTS inhibition and DPPH inhibition, is shown in Table 7.

The percentage of ABTS inhibition in the lucuma control was 39.62%, while for lucuma DIC-treated samples, it ranged from 35.73% to 47.96%. Comparing the control to DIC 5 (0.27 MPa and 22 s), this treatment increased the ABTS inhibition by 21%. However, DIC 8 (0.13 MPa and 8 s) reduced the ABTS inhibition by 10% relative to the control. Table 8 shows the analysis of variance of the antioxidant activity of SD lucuma.

**Figure 9:**
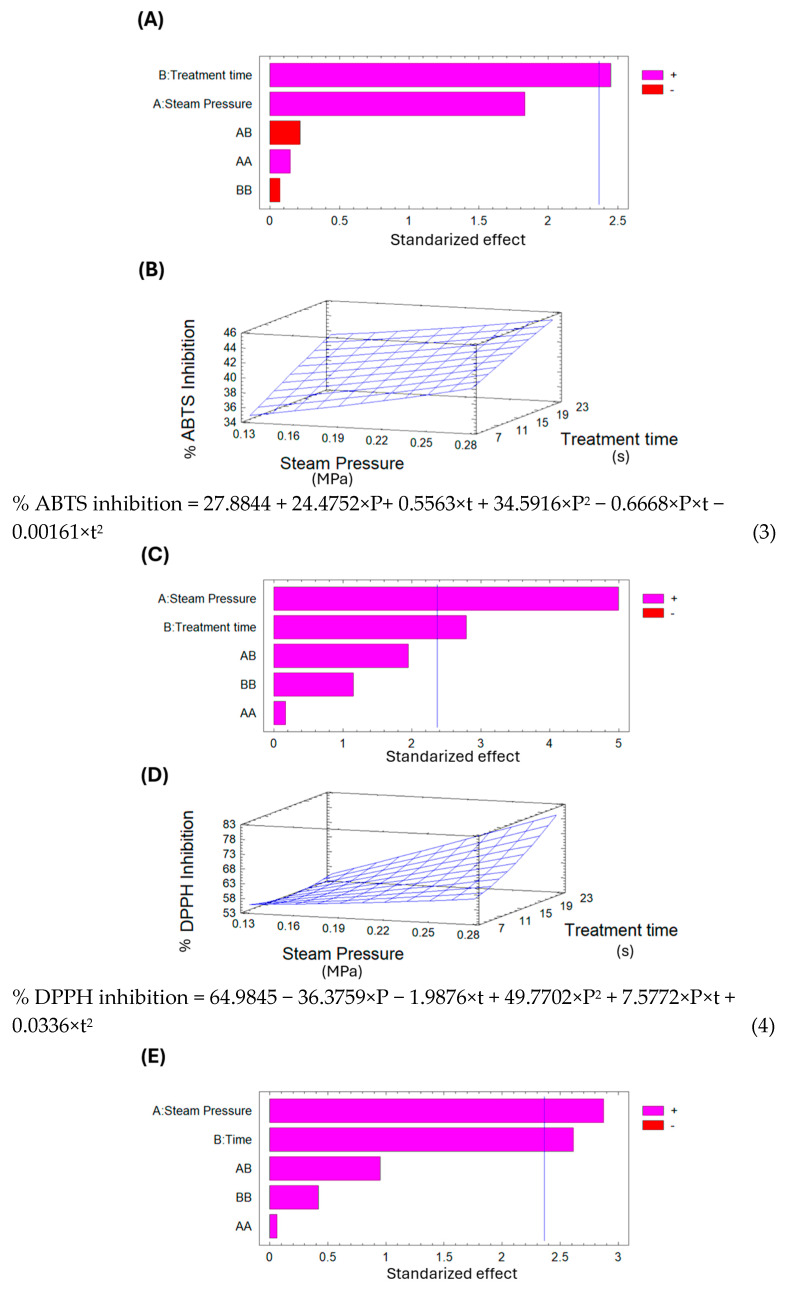

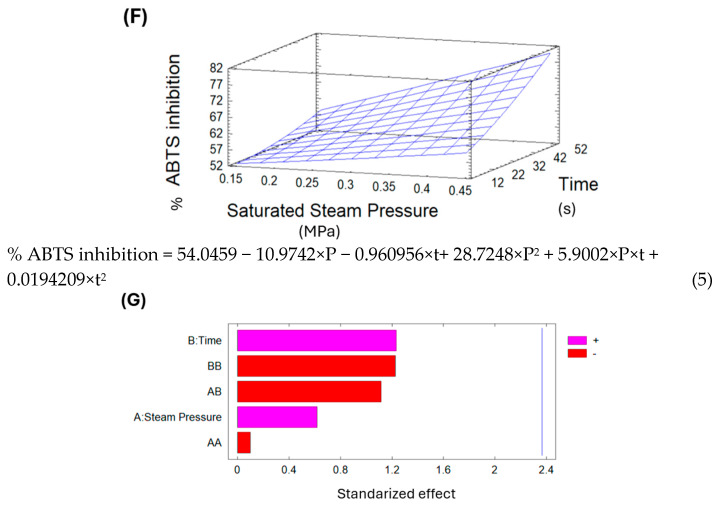
Antioxidant activity of DIC-treated lucuma and goldenberry. (**A**) Pareto chart of ABTS inhibition percentage of dried lucuma; (**B**) response surface of ABTS inhibition percentage of dried lucuma; (**C**) Pareto chart of DPPH inhibition percentage of dried lucuma; (**D**) response surface of DPPH inhibition percentage of dried lucuma; (**E**) Pareto chart of ABTS inhibition percentage of dried goldenberry; (**F**) response surface of ABTS inhibition percentage of dried goldenberry; and (**G**) Pareto chart of DPPH inhibition percentage of dried goldenberry.

The Pareto chart in Figure 9A shows that treatment time significantly affects the ABTS inhibition percentage. Additionally, the surface response in Figure 9B indicates that, under the selected parameters for DIC treatment, longer treatment times and higher saturated steam pressures lead to increased ABTS inhibition of dried lucuma. Using the surface response equation, the predicted optimal DIC conditions to maximize ABTS inhibition of pre-dried lucuma were 0.29 MPa and 25 s.

Conversely, analyzing the DPPH percentage inhibition of dried lucuma in Table 7 shows that the control sample had 49.22%, while the DIC samples ranged from 47.36% to 72.26%. DIC 11 (0.1 MPa and 15 s) exhibited the lowest inhibition percentage, 4% less than the control; in contrast, DIC 5 (0.27 MPa and 22 s) increased the DPPH inhibition percentage by 1.5 times. Additionally, the Pareto chart in Figure 9C indicates that both saturated steam pressure and thermal treatment time influence the DPPH inhibition percentage. Furthermore, the surface response graph in Figure 9D shows that, under the chosen DIC treatment parameters, the higher the saturated steam pressure and the longer the treatment time, the higher the DPPH inhibition in dried lucuma. Using the surface response model equation, the predicted optimal DIC conditions to maximize the percentage of DPPH inhibition in pre-dried lucuma can be set at 0.29 MPa and 25 s.

Among the reported antioxidants of lucuma, Aguilar-Galvez and García-Ríos [1] indicate that ripe lucuma is rich in carotenoids (0.15 mg β-carotene equivalent/g dw), phenols (69.3 mg gallic acid equivalent/g dw), sterols (6.5 μg β-sitosterol/g dw and 0.86 μg cycloartenol/g dw), α-amyrin (25.4 μg/g dw), myo-inositol (3.17 mg/g dw), and α-tocopherol (5.1 mg/100 g dw). In this regard, it can be noted that after swell-drying, the yellow-orange color of lucuma pulp was maintained, which may indicate good preservation of carotenoids. Therefore, future studies could analyze the impact of IHAD and DIC on each of these molecules and their relation to the antioxidant activity of dried lucuma.

Regarding untreated and DIC-treated goldenberries, Table 9 shows their antioxidant activity measured by the percentage of ABTS and DPPH inhibition.

The control goldenberry sample exhibited 56% ABTS inhibition, while DIC-treated samples ranged from 50.65% to 74.08%. The lowest ABTS inhibition among DIC samples was observed with DIC 11 (0.1 MPa for 30 s), whereas DIC 2 (0.5 MPa for 30 s) and DIC 3 (0.3 MPa for 55 s) increased the ABTS inhibition by 1.3 times. Table 10 shows the analysis of variance of the antioxidant activity of SD goldenberry.

The Pareto chart in Figure 9E indicates that both saturated steam pressure and treatment time influence the percentage of ABTS inhibition. Additionally, the surface response graph in Figure 9F shows that within the selected variable range for DIC treatment, higher saturated steam pressure and longer treatment times result in greater ABTS inhibition. Using the surface response model equation, the predicted optimal conditions to maximize ABTS inhibition in goldenberry are 0.49 MPa and 55 s. Naranjo-Durán and Quintero-Quiroz [54] state that goldenberry has a good capacity to trap free radicals. And Narváez-Cuenca and Mateus-Gómez [55] also observed higher ABTS values in dried goldenberry fruits processed with an airflow at 60 °C compared to fresh fruits. This increase in ABTS values could be linked to a good preservation of bioactive compounds and/or to the generation of Maillard products with a good radical scavenger activity.

Finally, regarding the DPPH percentage of inhibition of goldenberry, the control showed 75.91%, while DIC samples ranged from 58.02% to 79.6%. DIC 8 (0.16 MPa and 12 s) exhibited the lowest DPPH percentage of inhibition, 24% less than the control. In the Pareto chart of Figure 9G, none of the selected variables could explain the variations in DPPH percentage of inhibition. For this reason, it was not possible to present the surface response graph and the corresponding equation.

The study by İzli and Yıldız [56] indicates that by comparing the DPPH antioxidant activity of fresh fruit vs. dried goldenberry (convective, microwave, and microwave + convective drying), fresh samples had significantly higher antioxidant capacity than the dried samples. Conversely, DIC 11 (0.10 MPa, 30 s) showed the highest DPPH percentage of inhibition, 4.9% higher than the control. This slight increase could be linked to a good preservation of bioactive compounds such as phenolics. Yıldız and İzli [42] also studied the antioxidant capacity of fresh goldenberries, and they found a 57.67% inhibition of DPPH. On the other hand, Olivares-Tenorio and Verkerk [57] studied the antioxidant activity of rehydrated freeze-dried goldenberries heated from 40 to 120 °C, and their results showed that after heating (100 °C during 120 min), antioxidant activity was reduced from 547.6 to 355 μg Trolox Equivalent 100 g^−1^ FW. Moreover, among the several bioactive molecules responsible for the antioxidant activity of goldenberry, the study highlights ascorbic acid as the main molecule that contributes to DPPH inhibition.

Furthermore, the antioxidant capacity of goldenberry may be linked to the levels of phenols, flavonoids, and carotenoids. In fact, these compounds act as scavengers of free radicals produced during oxidation reactions [58,59]. Biasi and Huber [60] identified 23 bioactive compounds in goldenberry flour, including benzoic acid, chlorogenic acid, 2,4-dihydroxybenzoic acid, 2,5-dihydroxybenzoic acid, 3,4-dihydroxybenzoic acid, synaptic acid, ferulic acid, p-coumaric acid, caffeic acid, salicylic acid, synapaldehyde, syringaldehyde, syringic acid, pinocembrin, galangin, apigenin, kaempferol, epicatechin, catechin, hesperidin, quercetin, naringenin, and naringin. 

Olivares-Tenorio and Verkerk [57] also identified the presence of catechin, epicatechin, rutin, quercetin dihydrate, myricetin, and kaempferol in goldenberries; however, after heat treatment, only catechin and epicatechin were measured. Nocetti and Núñez [61] reported the presence of tocopherols and sterols in goldenberry, with β-tocopherol and campesterol being the most abundant. Furthermore, the study by Jéssica and Vega-Gálvez [62] showed that air drying goldenberry at 50 °C resulted in a 28% loss of β-carotene compared to the control, and drying at 90 °C showed no significant difference from the control. The authors observed the same pattern with total phenol content (TPC), where higher air-drying temperatures reduced the initial TPC in goldenberry, except at 90 °C. TPC increased from 321.05 to 356.68 mg gallic acid per 100 g of dry matter. These findings demonstrate that the bioavailability of antioxidant compounds can be influenced by various factors, such as the binding of phenolic compounds within the food matrix, differences in cell wall structures, and the location of bioactive compounds within cells—all directly related to fruit drying conditions. Therefore, it is possible that, after DIC treatment, the extractability of certain bioactive compounds was improved due to the formation of new porous structures during the process of autovaporization.

In fact, various studies have shown that adequate DIC treatment can improve the antioxidant activity of food products, such as beetroots, amaranth, okra, pepper, lentils, and cardamom [21,22,63,64,65,66]. During the instant controlled pressure drop step, the system immediately undergoes autovaporization, causing quick expansion, which creates a porous structure and rapidly cools biological matrices. This cooling protects thermosensitive compounds, and the porous structure enhances the extraction of active molecules, both significantly increasing the nutritional value of dried foods. Moreover, IHAD can prevent the degradation of thermosensitive molecules by significantly reducing the exposure time of fruits to high temperatures. This study also showed that DIC treatment could boost the antioxidant activity of dried lucuma and goldenberry, with IHAD offering an innovative pre-drying process to enhance the nutritional quality of dried fruits.

## 4. Conclusions

Swell-dried goldenberry and lucuma could boost Peru’s economy. In fact, applying swell-drying to both Andean fruits maintained their nutritional quality, and under accurate DIC treatments, antioxidant activity was increased compared to the controls. For lucuma, moisture content was reduced from 62.12% w.b. to a range between 7.98 and 11.8% w.b. Additionally, lipids ranged from 1.16 to 1.46% of DM, proteins from 7.42 to 9.01% of DM, fiber from 0.11 to 1.85% of DM, ash from 1.29 to 2.42% of DM, and carbohydrates from 86.72 to 91% of DM. The experimental swell-drying conditions to increase lucuma ABTS inhibition by 1.2 times and DPPH inhibition by 1.5 times were found after an initial pre-drying under Interval Highly Active Drying (IHAD) with an active time t_ON_ of 1.88 s and a tempering time t_OFF_ of 58.13 s, followed by a DIC treatment at 0.27 MPa for 22 s, and a final continuous convective post-drying.

On the other hand, in the case of goldenberry, moisture content decreased from 25.93% w.b to a range between 9.61% and 22.95% w.b. Since the skin acts as a barrier that limits water evaporation, the final moisture content of swell-dried samples depended on the applied DIC treatment. The DIC treatment of 0.30 MPa for 30 s facilitates moisture evaporation from inside the fruit, resulting in a final moisture content of 9.61%. Additionally, considering the macronutrient composition of dried goldenberry, it was observed that lipids ranged from 5.25 to 8.88% of DM, proteins from 5.15 to 8.61% of DM, fiber from 2.48 to 8.25% of DM, ash from 5.97 to 7.34% of DM, and carbohydrates from 69.4 to 77.25% of DM. Furthermore, the experimental swell-drying conditions for increasing the percentage of ABTS inhibition by 1.5 times were found under an initial continuous airflow convective pre-drying, followed by a DIC treatment of 0.5 MPa for 30 s. Moreover, the best swell-drying conditions for increasing the % of DPPH inhibition of goldenberry into 4.9% compared to the control were found after CCAD pre-drying, followed by a DIC treatment of 0.10 MPa for 30 s. For this response, none of the selected variables could fully explain the changes in DPPH inhibition percentage.

Finally, the application of swell-drying to Andean fruits has the potential to create significant economic and social benefits. From an economic perspective, this innovative technology preserves nutritional quality and reduces drying times. And from a social perspective, the valorization of native Andean crops could support sustainable rural development.

## 5. Perspectives

Future studies should evaluate the effect of IHAD on preserving the main bioactive compounds responsible for the antioxidant activity of lucuma and goldenberry, as well as the energy savings of this process compared to continuous drying. In addition, further research is needed to evaluate the effect of DIC treatment on the moisture permeability of goldenberries’ waxy skin to improve their industrial drying. Moreover, inasmuch as in this preliminary research, the experimental validation of the predicted DIC optimal operating conditions was not assessed. In a further study, it will be necessary to validate the thought experiments and the predicted optimized conditions, and to compare the predicted and experimental values to assess the reliability of the models. Finally, for both SD samples, the profile of bioactive compounds, the color, the sensory properties, and the assessment of food product storage and shelf life could be investigated to better identify the most adequate swell-drying conditions for each fruit.

## Figures and Tables

**Table 1:** Experimental design of DIC treatment of pre-dried lucuma.

Treatment Trials	Saturated Steam Pressure	Thermal Treatment Time
	MPa	s
DIC 1	0.20	15
DIC 2	0.30	15
DIC 3	0.20	25
DIC 4	0.20	15
DIC 5	0.27	22
DIC 6	0.27	8
DIC 7	0.20	15
DIC 8	0.13	8
DIC 9	0.13	22
DIC 10	0.20	15
DIC 11	0.10	15
DIC 12	0.20	5
DIC 13	0.20	15

**Table 2:** Experimental design for DIC treatment of pre-dried goldenberry.

Treatment Trials	Saturated Steam Pressure	Thermal Treatment Time
	MPa	s
DIC 1	0.30	30
DIC 2	0.50	30
DIC 3	0.30	55
DIC 4	0.30	30
DIC 5	0.44	48
DIC 6	0.44	12
DIC 7	0.30	30
DIC 8	0.16	12
DIC 9	0.16	48
DIC 10	0.30	30
DIC 11	0.10	30
DIC 12	0.30	5
DIC 13	0.30	30

**Table 3:** Results of the bromatological analysis of lucuma (*Pouteria lucuma*).

Sample	% Lipids	% Protein	% Fiber	% Ash	% Moisture	% Carbohydrates *
Control	1.19 ± 0.03	7.8 ± 0.5	0.1100 ± 0.0004	1.84 ± 0.07	9.69 ± 0.08	89.09
DIC 1	1.16 ± 0.03	9.0 ± 0.6	0.839 ± 0.012	1.54 ± 0.05	9.64 ± 0.14	87.45
DIC 2	1.24 ± 0.04	7.4 ± 0.6	0.498 ± 0.014	1.76 ± 0.17	9.36 ± 0.14	89.08
DIC 3	1.063 ± 0.012	7.9 ± 0.3	0.529 ± 0.016	2.33 ± 0.19	8.42 ± 0.11	88.21
DIC 4	1.05 ± 0.05	8.4 ± 0.5	0.87 ± 0.04	2.25 ± 0.12	10.0 ± 0.2	87.44
DIC 5	1.02 ± 0.06	6.1 ± 0.3	1.06 ± 0.02	1.61 ± 0.12	9.1 ± 0.5	90.19
DIC 6	0.931 ± 0.015	6.0 ± 0.6	0.2211 ± 0.0013	1.87 ± 0.06	9.26 ± 0.15	91.00
DIC 7	1.46 ± 0.05	5.1 ± 0.4	1.162 ± 0.012	1.781 ± 0.004	11.69 ± 0.03	90.46
DIC 8	1.30 ± 0.06	6.9 ± 0.4	1.628 ± 0.011	1.29 ± 0.13	11.80 ± 0.07	88.88
DIC 9	1.16 ± 0.05	6.5 ± 0.5	0.22 ± 0.02	1.72 ± 0.04	10.61 ± 0.14	90.44
DIC 10	0.97 ± 0.04	7.7 ± 0.5	0.40 ± 0.03	2.420 ± 0.002	11.4 ± 0.3	88.51
DIC 11	1.32 ± 0.12	6.91 ± 0.12	1.80 ± 0.08	2.369 ± 0.011	8.0 ± 0.2	87.60
DIC 12	1.38 ± 0.10	7.7 ± 0.8	1.85 ± 0.14	1.91 ± 0.09	8.9 ± 0.6	87.18
DIC 13	1.30 ± 0.02	8.6 ± 0.5	1.74 ± 0.12	1.65 ± 0.06	7.98 ± 0.04	86.73

Data are expressed in g/100 g of dry matter. All values represent the mean ± standard deviation in duplicate. * Carbohydrates were calculated by difference.

**Table 4:** Analysis of variance for bromatological analysis of SD lucuma (*Pouteria lucuma*).

**% Lipids**					
**Source**	**Sum of Squares**	**DDL**	**Mean Square**	**Ratio F**	**Probability**
A: Steam pressure	0.0485	1	0.0485	1.4300	0.2705
B: Treatment time	0.0316	1	0.0316	0.9300	0.3667
AA	0.0006	1	0.0006	0.0200	0.8997
AB	0.0132	1	0.0132	0.3900	0.5521
BB	0.0030	1	0.0030	0.0900	0.7736
Total error	0.2373	7	0.0339		
R^2^	0.2908				
% Protein					
Source	Sum of Squares	DDL	Mean Square	Ratio F	Probability
A: Steam pressure	0.0363	1	0.0363	0.0200	0.8906
B: Treatment time	0.0001	1	0.0001	0.0000	0.9936
AA	2.3060	1	2.3060	1.2900	0.2927
AB	0.0841	1	0.0841	0.0500	0.8342
BB	0.5100	1	0.5100	0.2900	0.6092
Total error	12.4729	7	1.7818		
R^2^	0.1778				
% Fiber					
Source	Sum of Squares	DDL	Mean Square	Ratio F	Probability
A: Steam pressure	0.7251	1	0.7251	3.0100	0.1264
B: Treatment time	0.7422	1	0.7422	3.0800	0.1227
AA	0.0036	1	0.0036	0.0200	0.9057
AB	1.2656	1	1.2656	5.2500	0.0557
BB	0.0001	1	0.0001	0.0000	0.9881
Total error	1.6873	7	0.2410		
R^2^	0.6185				
% Ash					
Source	Sum of Squares	DDL	Mean Square	Ratio F	Probability
A: Steam pressure	0.0193	1	0.0193	0.1100	0.7551
B: Treatment time	0.0730	1	0.0730	0.4000	0.5479
AA	0.0167	1	0.0167	0.0900	0.7714
AB	0.1190	1	0.1190	0.6500	0.4466
BB	0.0032	1	0.0032	0.0200	0.8983
Total error	1.2818	7	0.1831		
R^2^	0.1519				
% Moisture					
Source	Sum of Squares	DDL	Mean Square	Ratio F	Probability
A: Steam pressure	0.6100	1	0.6100	0.2400	0.6423
B: Treatment time	0.5061	1	0.5061	0.2000	0.6718
AA	0.8316	1	0.8316	0.3200	0.5886
AB	0.2450	1	0.2450	0.0900	0.7673
BB	0.9692	1	0.9692	0.3700	0.5600
Total error	18.1283	7	2.5898		
R^2^	0.1401				
% Carbohydrates					
Source	Sum of Squares	DDL	Mean Square	Ratio F	Probability
A: Steam pressure	1.9632	1	1.9632	0.7900	0.4048
B: Treatment time	0.6087	1	0.6087	0.2400	0.6367
AA	2.8538	1	2.8538	1.1400	0.3206
AB	1.4042	1	1.4042	0.5600	0.4778
BB	0.7035	1	0.7035	0.2800	0.6121
Total error	17.4852	7	2.4979		
R^2^	0.2922				

Analysis of variance with *p* ≤ 0.05.

**Table 5:** Results of the bromatological analysis of goldenberry (*Physalis peruviana* L.).

Sample	% Lipids	% Protein	% Fiber	% Ash	% Humidity	% Carbohydrates *
Control	5.41 ± 0.06	6.88 ± 0.16	7.5 ± 0.2	7.0 ± 0.3	20.39 ± 0.02	73.14
DIC 1	5.3 ± 0.4	6.8 ± 0.3	8.3 ± 0.4	6.9 ± 0.3	19.40 ± 0.05	72.84
DIC 2	5.94 ± 0.09	8.61 ± 0.18	7.05 ± 0.10	6.3 ± 0.2	19.2 ± 0.6	72.07
DIC 3	6.54 ± 0.14	7.76 ± 1.13	2.48 ± 0.18	5.97 ± 0.07	18.49 ± 0.06	77.25
DIC 4	8.2 ± 0.6	7.64 ± 0.18	4.7 ± 0.3	6.3 ± 0.6	19.5 ± 0.5	73.18
DIC 5	7.8 ± 0.3	7.7 ± 0.3	6.52 ± 0.09	6.9 ± 0.2	19.1 ± 0.5	71.08
DIC 6	8.84 ± 0.06	7.54 ± 0.05	5.47 ± 0.10	6.8 ± 0.4	19.2 ± 0.6	71.32
DIC 7	5.9 ± 0.4	7.4 ± 0.2	7.30 ± 0.14	6.6 ± 0.4	9.6 ± 0.6	72.82
DIC 8	6.8 ± 0.6	7.46 ± 0.18	7.8 ± 0.2	7.1 ± 0.5	11.1 ± 0.3	70.90
DIC 9	6.3 ± 0.4	6.9 ± 0.2	5.92 ± 0.08	6.2 ± 0.4	18.5 ± 0.4	74.66
DIC 10	8.19 ± 0.17	7.4 ± 0.3	7.0 ± 0.2	7.3 ± 0.3	18.9 ± 0.4	70.08
DIC 11	8.4 ± 0.8	6.1 ± 0.3	6.9 ± 0.4	7.20 ± 0.02	19.3 ± 0.4	71.42
DIC 12	8.5 ± 0.7	5.15 ± 1.12	7.9 ± 0.6	7.13 ± 0.06	21.6 ± 0.5	71.38
DIC 13	6.27 ± 0.18	7.34 ± 0.12	7.7 ± 0.4	7.1 ± 0.4	23.0 ± 1.3	71.66

Data are expressed in g/100 g of dry matter. All values represent the mean ± standard deviation in duplicate. * Carbohydrates were calculated by difference.

**Table 6:** Analysis of variance for bromatological analysis of SD lucuma.

**% Lipids**					
**Source**	**Sum of Squares**	**DDL**	**Mean Square**	**Ratio F**	**Probability**
A: Steam pressure	0.003	1	0.003	0.000	0.972
B: Treatment time	2.154	1	2.154	1.110	0.328
AA	0.336	1	0.336	0.170	0.690
AB	0.093	1	0.093	0.050	0.833
BB	1.085	1	1.085	0.560	0.480
Total error	13.637	7	1.948		
R^2^	0.206				
% Protein					
Source	Sum of Squares	DDL	Mean Square	Ratio F	Probability
A: Steam pressure	2.412	1	2.412	4.420	0.074
B: Treatment time	1.354	1	1.354	2.480	0.159
AA	0.178	1	0.178	0.330	0.586
AB	0.137	1	0.137	0.250	0.632
BB	0.627	1	0.627	1.150	0.320
Total error	3.823	7	0.546		
R^2^	0.557				
% Fiber					
Source	Sum of Squares	DDL	Mean Square	Ratio F	Probability
A: Steam pressure	0.290	1	0.290	0.150	0.713
B: Treatment time	9.033	1	9.033	4.560	0.070
AA	0.042	1	0.042	0.020	0.888
AB	2.176	1	2.176	1.100	0.330
BB	4.618	1	4.618	2.330	0.171
Total error	13.881	7	1.983		
R^2^	0.541				
% Ash					
Source	Sum of Squares	DDL	Mean Square	Ratio F	Probability
A: Steam pressure	0.074	1	0.074	0.460	0.521
B: Treatment time	0.757	1	0.757	4.650	0.068
AA	0.001	1	0.001	0.010	0.940
AB	0.212	1	0.212	1.300	0.292
BB	0.099	1	0.099	0.610	0.461
Total error	1.140	7	0.163		
R^2^	0.500				
% Moisture					
Source	Sum of Squares	DDL	Mean Square	Ratio F	Probability
A: Steam pressure	8.933	1	8.933	0.440	0.527
B: Treatment time	1.024	1	1.024	0.050	0.828
AA	0.054	1	0.054	0.000	0.960
AB	14.364	1	14.364	0.710	0.427
BB	0.708	1	0.708	0.040	0.857
Total error	141.098	7	20.157		
R^2^	0.151				
% Carbohydrates					
Source	Sum of Squares	DDL	Mean Square	Ratio F	Probability
A: Steam pressure	0.013	1	0.013	0.000	0.957
B: Treatment time	6.602	1	6.602	1.640	0.241
AA	6.126	1	6.126	1.520	0.257
AB	0.048	1	0.048	0.010	0.916
BB	4.442	1	4.442	1.100	0.328
Total error	28.157	7	4.022		
R^2^	0.400				

Analysis of variance with *p* ≤ 0.05.

**Table 7:** Impact of DIC treatment on the antioxidant capacity of lucuma methanolic extract.

Sample	Treatment Conditions		Response Variables	
	Saturated Steam Pressure	Thermal Treatment Time	ABTS	DPPH
	(MPa)	(s)	% Inhibition	% Inhibition
Control	NA	NA	39.6 ± 0.8	49.2 ± 0.9
DIC 1	0.2	15	44.189 ± 3.011	61.1 ± 0.8
DIC 2	0.3	15	41.1 ± 2.2	69.5 ± 0.9
DIC 3	0.2	25	40.0 ± 2.5	67.2 ± 0.3
DIC 4	0.2	15	39.2 ± 2.3	60.0 ± 1.5
DIC 5	0.27	22	48.0 ± 2.7	77.3 ± 0.5
DIC 6	0.27	8	40.8 ± 1.7	62.9 ± 0.4
DIC 7	0.2	15	38.3 ± 2.4	65.4 ± 2.1
DIC 8	0.13	8	35.7 ± 2.3	58.9 ± 1.9
DIC 9	0.13	22	44.3 ± 1.8	58.2 ± 0.6
DIC 10	0.2	15	40.1 ± 1.9	55.1 ± 0.3
DIC 11	0.1	15	36.0 ± 1.3	47.4 ± 0.7
DIC 12	0.2	5	36.1 ± 1.8	55.4 ± 0.9
DIC 13	0.2	15	38.9 ± 4.2	59.3 ± 0.2

NA: not applied.

**Table 8:** Analysis of variance for the antioxidant capacity of SD lucuma.

**ABTS % Inhibition**					
**Source**	**Sum of Squares**	**DDL**	**Mean Square**	**Ratio F**	**Probability**
A: Steam pressure	31.4167	1	31.4167	3.3500	0.1098
B: Treatment time	56.1429	1	56.1429	5.9900	0.0443
AA	0.1999	1	0.1999	0.0200	0.8880
AB	0.4356	1	0.4356	0.0500	0.8355
BB	0.0451	1	0.0451	0.0000	0.9466
Total error	65.6065	7	9.3724		
R^2^	0.5736				
DPPH % Inhibition					
Source	Sum of Squares	DDL	Mean Square	Ratio F	Probability
A: Steam pressure	370.2910	1	370.2910	24.9200	0.0016
B: Treatment time	115.2930	1	115.2930	7.7600	0.0271
AA	0.4137	1	0.4137	0.0300	0.8722
AB	56.2500	1	56.2500	3.7900	0.0928
BB	19.6662	1	19.6662	1.3200	0.2878
Total error	104.0200	7	14.8600		
R^2^	0.8437				

Analysis of variance with *p* ≤ 0.05.

**Table 9:** Impact of DIC treatment on the antioxidant capacity of goldenberry (*Physalis peruviana* L.) methanolic extract.

Sample	Treatment Conditions		Response Variables	
	Saturated Steam Pressure	Thermal Treatment Time	ABTS	DPPH
	(MPa)	(s)	% Inhibition	% Inhibition
Control	NA	NA	56.8 ± 0.7	75.9 ± 0.7
DIC 1	0.30	30	72.9 ± 5.7	76.3 ± 0.6
DIC 2	0.50	30	73.6 ± 2.7	75.3 ± 2.5
DIC 3	0.30	55	74.1 ± 2.6	70.6 ± 2.9
DIC 4	0.30	30	57.06 ± 1.06	78.78 ± 3.04
DIC 5	0.44	48	71.9 ± 3.4	78.69 ± 2.12
DIC 6	0.44	12	57.9 ± 0.5	72.3 ± 0.8
DIC 7	0.30	30	54.7 ± 0.9	74.33 ± 0.98
DIC 8	0.16	12	55.1 ± 0.6	58.0 ± 4.45
DIC 9	0.16	48	57.4 ± 1.8	77.0 ± 1.8
DIC 10	0.30	30	58.3 ± 0.7	74.0 ± 1.2
DIC 11	0.10	30	50.7 ± 1.2	79.6 ± 1.9
DIC 12	0.30	5	53.5 ± 0.7	74.7 ± 0.6
DIC 13	0.30	30	60.3 ± 2.2	77.17 ± 1.13

NA: not applied.

**Table 10:** Analysis of variance for the antioxidant capacity of SD goldenberry.

**ABTS % Inhibition**					
**Source**	**Sum of Squares**	**DDL**	**Mean Square**	**Ratio F**	**Probability**
A: Steam pressure	310.632	1	310.632	8.240	0.024
B: Treatment time	257.016	1	257.016	6.820	0.035
AA	0.138	1	0.138	0.000	0.954
AB	34.106	1	34.106	0.900	0.373
BB	6.556	1	6.556	0.170	0.689
Total error	263.879	7	37.697		
R^2^	0.697				
DPPH % Inhibition					
Source	Sum of Squares	DDL	Mean Square	Ratio F	Probability
A: Steam pressure	12.195	1	12.195	0.380	0.556
B: Treatment time	48.286	1	48.286	1.520	0.258
AA	0.320	1	0.320	0.010	0.923
AB	39.627	1	39.627	1.250	0.301
BB	47.825	1	47.825	1.500	0.260
Total error	222.794	7	31.828		
R^2^	0.399				

## Data Availability

All related data and methods are presented in this paper. Additional inquiries should be addressed to the corresponding author.

## Abbreviations

The following abbreviations are used in this manuscript:

SD	Swell-drying
IHAD	Interval Highly Active Drying
DIC	Instant Controlled Pressure Drop
CCAD	Continuous Convective Airflow Drying
